# Polypharmacy and drug interactions in older patients with cancer receiving chemotherapy: associated factors

**DOI:** 10.1186/s12877-024-05135-6

**Published:** 2024-06-25

**Authors:** Rita F. Oliveira, Ana I. Oliveira, Agostinho S. Cruz, Oscar Ribeiro, Vera Afreixo, Francisco Pimentel

**Affiliations:** 1 University of Aveiro, Aveiro, Portugal; 2 ESS, Polytechnic of Porto, Porto, Portugal; 3 Center for Health Technology and Services Researchat the Associate Laboratory RISE – Health Research Network (CINTESIS@RISE), Department of Education and Psychology, University of Aveiro (UA), Aveiro, Portugal; 4 REQUIMTE/LAQV, ESS, Polytechnic of Porto, Porto, Portugal; 5 Center for Research and Development in Mathematics and Applications (CIDMA), Department of Mathematics, University of Aveiro (UA), Aveiro, Portugal; 6 BlueClinical, Matosinhos, Portugal

**Keywords:** Polypharmacy, Drug interactions, Severe drug interactions, Antineoplastic agents, Elderly cancer patients

## Abstract

**Background:**

Polypharmacy in older adults with cancer receiving chemotherapy leads to increased risks of drug interactions, translating in potential hazardous health outcomes. This study aims to assess the prevalence of polypharmacy, drug–drug interactions (DDIs), and severe-drug interactions (SDIs) in older patients with cancer. Antineoplastic agents (ANAs) involvement and possible risk contexts (comorbidities with cardiac risk, and high-risk medications) were also analysed.

**Methods:**

Observational study with older adults (≥ 65 years) diagnosed with cancer, who were treated with antineoplastic agents (ANAs); it was conducted in three hospitals from the north of Portugal. Data collection was obtained using self-reports and medical records. DDIs were identified and classified using Micromedex® software. Descriptive and association analyze statistics were performed. Statistical hypothesis tests with p value less than 0.05 were considered significant. All statistical procedures and analysis were performed with R version 4.1.3.

**Results:**

We enrolled 552 patients. Polypharmacy prevalence was 88.40%; 76.45% and 56.16% of the patients presented with DDIs and SDIs, respectively. SDIs with ANAs were found in 21.20% of the patients. High-risk medications were associated with a higher risk of polypharmacy, DDIs, and SDIs. Polypharmacy and DDIs were higher in patients with hypertension or diabetes. SDIs were higher in patients with diabetes.

**Conclusion:**

Polypharmacy, potential DDIs and SDIs were highly prevalent in older adults with cancer. A careful review of the medication administered is necessary to decrease it. These findings warrant further research to optimize medication in this population and decrease problems related to medication, which may lead to emergency room visits and hospitalisations, compromising patient safety and/or ongoing treatments.

## Introduction

In the last few years, there has been an increase in cancer incidence in many countries, which is primarily ascribed to a significant increase in the senior population, with a prediction that by 2040, 47% of all new cancer diagnoses will be in adults aged ≥ 70 years [1]. Age is one of the main risk factors for cancer due to biological changes associated with the aging process [1–5].

Although older patients with cancer have heterogeneous clinical profiles, high comorbidities burden and polypharmacy (use of 5 or more medications) are common in this population [6–10], which translates into an important public health problem [11, 12].

Balducci, et al*.* (2011) reported that 35% of cancer patients aged ≥ 70 years use five or more medications at diagnosis prior to receiving anticancer treatment and supportive agents [10]. In more recent studies, the prevalence of polypharmacy in this population has been reported as between 45.2% and 90.8%, and excessive polypharmacy (use of 10 or more medications) has been identified, with variations between 8.6% and 18.2% [13–16]. Studies have revealed that polypharmacy is a highly prevalent condition in the elderly population, including in the Portuguese one [17, 18].

As a fragile population and being more vulnerable to adverse drug effects, geriatric patients with cancer undergoing chemotherapy tend to be more exposed to the risks of polypharmacy than the rest of the population [19–21]. This is because systemic anti-cancer therapies contribute to an additional drug burden in patients who are already taking multiple drugs for chronic diseases and may need supplementary supportive care medications, placing patients at increased risk of drug-related problems, such as drug-drug interactions (DDIs).

Drug interactions can be defined as the pharmacological or clinical events, in which the intended therapeutic effect or safety of a medication is altered by the administration of another substance. DDIs might result in an intensified effect of a drug, causing an increased risk of adverse events or reduce the effect of other drugs, leading to treatment failure [14, 22–25]. The most critical DDIs are the severe drug interactions (SDIs) because they have the potential to produce serious adverse clinical consequences and cause permanent damage should therefore be avoided [13, 16, 26].

Polypharmacy and DDIs are associated with an increase in adverse drug reactions [13, 15, 27, 28], treatment toxicity [29, 30], hospital admissions [28–30], falls, frailty [31, 32] and mortality [20, 33]. In older patients, polypharmacy is also associated with an increased risk of potentially inappropriate medications [15, 34].

Drug interactions may be highly prevalent in the geriatric oncology population, especially in patients undergoing chemotherapy. The risk of potential DDIs has been reported to range from 51% to 76.5% in this population [35–37], with SDIs reportedly ranging from 30.6% to 61.3% [13, 14, 16, 26]. In some studies, DDIs involving antineoplastic agents (ANAs), were between 26.4% and 45.9% [15, 35, 36].

Several factors predispose older adults with cancer to an increased risk of DDIs. The lack of coordination among the different professionals and, sometimes, the absence of guidelines or recommendations for managing certain diseases, the prescribing cascades and communication failures between patients and health professionals are additional factors in the high prevalence of DDIs in this population [14, 38].

Presently, there is limited information on polypharmacy and DDIs in older adults with cancer, and its possible relation with the existence of cardiovascular risk factors or the administration of high-risk medication. Cardiovascular diseases and cancer are still the leading causes of death, and their coexistence is common. High-risk medication includes medicines with an elevated risk of causing significant patient harm if not taken the right way, or that negatively interact with other drugs when taken together. The administration of these drugs has been associated with increased adverse drug events and risk of hospitalisations [13, 39, 40].

As this population is more prone to the occurrence of adverse reactions, studies are important to identify the factors associated with polypharmacy, DDIs and SDIs, to guide prevention measures and support treatment decisions in elderly cancer patients.

The aim of this study was to investigate the prevalence of polypharmacy, potential DDIs, and SDIs in older adults with cancer receiving chemotherapy and to identify its associated factors. The most frequent SDIs and the involvement of ANAs were also assessed. This study also aimed to identify the patterns associated with polypharmacy, DDIs, and SDIs, in different contexts: (I) administration of high-risk medications, and (ii) existence of comorbidities with cardiac risk (hypertension, diabetes mellitus or dyslipidemia). The insights from this study are expected to provide the overall therapeutic profile of this population.

## Materials and methods

### Study design

This is an analytical and cross-sectional study held in three hospitals in Porto, in northern Portugal. This study was conducted over a period of 16 months and included 552 participants. The study was approved by the Health Ethics Committees of each hospital institutions (Centro Hospitalar do Porto, Centro Hospitalar São João and Instituto Português de Oncologia), and informed consent was obtained from all patients prior to inclusion in the study.

### Participants and eligibility

**Fig. 1:**
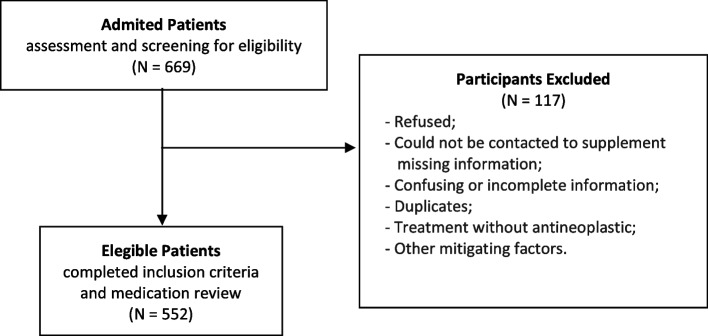
Flow diagram of participant screening

This study included older adults with a diagnosis of cancer. Patients aged 65 years or older undergoing chemotherapy, treated with ANAs, and with no cognitive impairment were eligible. Cognitive status was assessed using the Six-item Cognitive Impairment Test (6CIT) (Apóstolo et al., 2018). During the data collection period, all patients undergoing chemotherapy who met the inclusion criteria were invited to take part in the study. The exclusion criteria included patients who did not master the Portuguese language or who were not responsible for managing their own medication. Patients with incomplete data were excluded from the analysis (Fig. 1). A non-probabilistic sampling for convenience was performed, in which the sample size was calculated using EpiInfo™® (Version 7.1.5/2015).

### Data collection

Data collection was conducted using a structured questionnaire applied to all participants during the chemotherapy treatment.

The collected data included standard demographic information, medical conditions, comorbidities (including diabetes mellitus, hypertension, and dyslipidemia), and a detailed list of all medications administered. Information about medication use was obtained using both self-reports and by consulting each patient’s medical records. Where necessary, telephone contact supplemented the information. Additional information such as the type of cancer and antineoplastic treatment were obtained from the patient's clinical process.

### Outcome measurements

In this study, polypharmacy was defined as the use of five or more medications. The use of ten or more medications was labelled excessive polypharmacy [13, 29, 33, 41, 42].

Potential drug interaction was assessed using the Micromedex® software (electronic version) [43], that classifies DDIs into five severity levels: contraindicated, major, moderate, minor, and unknown. The most valued DDIs and clinically relevant were SDIs, which included major and contraindicated interactions. Micromedex® is based on published data and shows superior precision, comprehensive, sensitivity, and specificity when compared to other drug interactions resources. This makes it interesting for use in routine clinical practice, particularly when performing reviews of medication in patients taking multiple drugs. Micromedex® solutions database has been used in other oncology drug interaction studies [44, 45].

Drugs were classified according to the Anatomical Therapeutic Chemical (ATC) classification system codes. The oncological context of the patients is coded by the International Statistical Classification of Diseases and Related Health Problems 10th Revision (ICD-10)—WHO (Version 2019). High-risk medications were identified according to the following categories: anticoagulants, antiplatelet agents, insulin, oral hypoglycemic agents, opioids, and antiarrhythmic drugs [13, 39].

### Statistical analysis

The data were summarised by location measures (mean, median, minimum, and maximum) and dispersion measures (standard error and range). The variables under study presented a non-gaussian distribution. Quantitative variables were analysed through the Wilcoxon-Mann–Whitney Test, qualitative variables were analysed with Pearson’s chi-square test, and association between two quantitative variables were evaluated with Spearman correlation test (and described by corresponding correlation coefficient). Univariate analyses were conducted, followed by multivariable analysis, using logistic regression models. The association effect sizes were measured as odds ratio (OR). All statistical procedures and analysis were performed with R version 4.1.3. Statistical hypothesis tests with p-values less than 0.05 were considered significant. Confidence intervals are reported with a 95% confidence level.

## Results

### Baseline characteristics

A total of 552 patients were included in this study, of which 308 were male (56%). The median age was 71 years (range: 65–89), with 9% of the patients being older than 80 years. The mean age was 71.88 years (SD = 5.04). The baseline characteristics are summarised in Table 1.

**Table 1:** Baseline characteristics of the enrolled patients (*n* = 552)

Variable	n	%
**Age, median (range), years**	71 (65–89)	
**Age, mean (SD)**	71.88 (5.04)	
65–79	503	91.12%
> 80	49	8.88%
**Sex**		
Male	308	55.69%
Female	244	44.31%
**Comorbidities**		
No	64	11.59%
Yes	488	88.41%
≥ 2	332	60.14%
**Comorbidities / Diseases**		
Heart	353	63.95%
Endocrine	143	25.91%
Osteoarticular	109	19.75%
Visual	85	15.40%
Digestive	78	14.13%
Respiratory	65	11.78%
Neurological	56	10.14%
Other	240	43.25%
**Comorbidities with cardiac risk (at least one)**	367	66.49%
Diabetes mellitus	125	22.64%
Hypertension	298	53.99%
Dyslipidemia	215	38.95%
**Cancer type** (ICD-10)		
Malignant neoplasms of digestive organs	200	36.23%
Malignant neoplasms of respiratory and intrathoracic organs	88	15.94%
Malignant neoplasm of breast	88	15.94%
Malignant neoplasms of male genital organs and urinary tract	60	10.87%
Malignant neoplasms, stated or presumed to be primary, of lymphoid, haematopoietic and related tissue	37	6.70%
Other tumours	36	6.52%
Malignant neoplasms of female genital organs	24	4.35%
Malignant neoplasms of bone and articular cartilage	10	1.81%
Malignant neoplasm of other and ill-defined sites: head, face and neck and malignant neoplasm of brain	5	0.91%
Melanoma and other malignant neoplasms of skin	4	0.72%

Regarding chronic diseases, 88.41% of the sample (*N* = 488) had at least one chronic disease, and 60.14% (*N* = 332) had more than two. Other common non-cancer diagnoses included hypertension (53.99%), dyslipidemia (38.95%), and diabetes mellitus (22.64%), in which 66.49% of the patients had at least one of the diseases (*N* = 367). The most common cancer types were “digestive system tumours” (36.23%), “lung, pleural, and thymic tumours” (15.94%), and “breast tumours” (15.94%).

### Medications and polypharmacy

According to the ATC classification system, and as shown in Table 2, most patients were administered drugs for acid related disorders (including antacids and drugs for peptic ulcer and gastro-oesophageal reflux disease [GORD]) (50%, *N* = 277); agents acting on the renin-angiotensin system (41%, *N* = 227); and lipid modifying agents (HMG CoA reductase inhibitors were included) (31%, *N* = 173); psycholeptics (30%, *N* = 165); analgesics (including opioids and antipyretics) (29%, *N* = 162), and drugs for diabetes mellitus (20%, *N* = 108). Drug groups administered to the patients were not homogeneous (Chi-square; *p*-value < 0.001).

**Table 2:** Therapeutic profile of patients

**Anatomical Therapeutic Chemical (ATC) Classification System**	Frequency	
	n	%
Drugs for acid related disorders	277	50.18%
Agents acting on the renin-angiotensin system	227	41.12%
Lipid modifying agents	173	31.34%
Psycholeptics	165	29.89%
Analgesics	162	29.34%
Drugs used in diabetes	108	19.57%
**Medication administered at home**		
For comorbidities and oncological context	312	56.52%
Only for comorbidities	145	26.27%
Only supportive medication	79	14.31%
Administration is unknown	16	2.90%
**High-risk medication**		
No	286	51.81%
Yes	266	48.19%
**High-risk medication**		
Anticoagulants or antiplatelets	131	23.73%
Oral hypoglycemic agents or insulins	109	19.74%
Opioids	88	15.94%
Antiarrhythmic	6	1.09%

Of the entire medication used, 57% represented drugs administered outside the cancer treatment context. A total of 266 (48%) patients used high-risk medications, with the most used in this category being anticoagulants/antiplatelets (24%; *N* = 131) and oral hypoglycemic agents/insulins (20%; *N* = 109).

The median number of medications administered per patient was 9 (range: 1–26), and the mean was 9.43 (SD = 4.37). The prevalence of polypharmacy was 88.40% (*N* = 488). Excessive polypharmacy was detected in 44.57% (*N* = 246) of the patients (Table 3).

**Table 3:** Medication, polypharmacy, DDIs, and SDIs

Variable	Frequency	
	n	%
**Number of medications**		
Mean, median (SD, range)	9.43, 9.00 (4.37, 1–26)	
0–4	64	11.59%
≥ 5	488	88.40%
≥ 10	246	44.57%
**Potential DDIs**		
Total number of DDIs	**1818**	
Mean	3.29	
Median (range)	2.00 (0–20)	
**DDIs – Severity (Micromedex®)**		
Major DDIs	**991**	**54.51%**
Moderate DDIs	758	41.69%
Minor DDIs	39	2.15%
DDIs Contraindicated	**30**	**1.65%**
**SDIs (major and contraindicated)**	**1021**	**56.16%**
**SDIs—Documentation (Micromedex®)**		
Excellent	56	5.48%
Good	150	14.69%
Fair	815	79.82%
**DDIs involving ANA**	**394**	**21.67%**
**SDIs involving ANA**	189	10.40%
**DDIs involving two ANA**	**49**	**2.70%**
**SDIs involving two ANA**	41	2.26%
**Patients exposed to DDIs** (*N* = 552)	**422**	**76.45%**
Major DDIs	308	55.80%
Moderate DDIs	340	61.59%
Minor DDIs	36	6.52%
Contraindicated DDIs	26	4.71%
**Patients exposed to SDIs** (*N* = 552) (major or contraindicated)	**310**	**56.16%**
**Patients exposed to DDIs involving ANA** (*N* = 552)	**225**	**40.76%**
**Patients exposed to SDIs involving ANA** (*N* = 552)	117	21.20%
**Patients exposed DDIs involving two ANA** (*N* = 552)	**45**	**8.15%**
**Patients exposed SDIs involving two ANA** (*N* = 552)	40	7.25%

*Abbreviations: DDIs* Drug–drug interactions, *SDIs* Severe drug interactions, *ANA* Antineoplastic agent

### Potential interactions

**Fig. 2:**
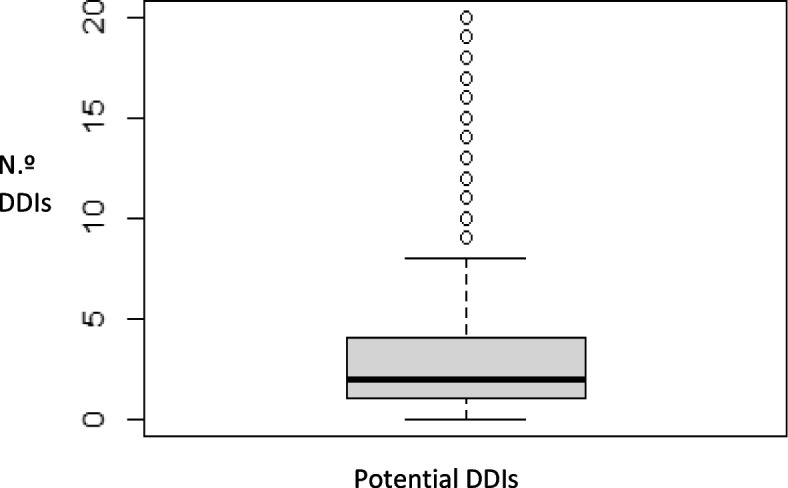
Boxplot of potential DDIs identified

A total of 1818 potential DDIs were identified. At least one potential DDI was identified in 422 participants (76.45%), with a mean of 3.29 and a median of 2.00 (range: 0–20) per patient (Fig. 2). The potential DDIs showed great variability, with 798 different DDIs identified. Of the total identified DDIs, 54.51% corresponded to major (*N* = 991), 41.69% to moderate (*N* = 758), 2.15% to minor (*N* = 39) and 1.65% to contraindicated (*N* = 30) DDIs.

Clinically significant interactions, SDIs (*N* = 1021; 56.16%), were identified in 56.16% of the patients (*N* = 310). Of the DDIs detected, 21.67% involved ANAs, and were identified in 40.76% of the patients (*N* = 225). Of all SDIs identified, 18.51% involved ANAs, and were identified in 21.20% of the patients (*N* = 117). Of the interactions involving ANAs, 47.97% corresponded to SDIs, which were detected in 52% of the patients. The most common potential SDIs identified are listed in Table 4.

**Table 4:** More frequent potential SDIs and potential outcomes in patients who had at least one DDI

Potential SDIs [43]	N	%	Involving ANA	Potential outcomes	Probable Mechanism [43]
Cyclophosphamide—doxorubicin	34	8.06%	Yes	Concurrent use of cyclophosphamide and doxorubicin may result in increased risk of cardiomyopathy and should be avoided and should be avoided and should be avoided. SDIs most frequently involve ANAs. Although no formal drug interaction studies have been done with doxorubicin hydrochloride liposome injection, it may interact with drugs known to interact with the conventional formulation of doxorubicin [46]	Additive or synergistic myocardial damage
Metoclopramide—tramadol Metoclopramide—lorazepam Metoclopramide—morphine Metoclopramide—diazepam	26 24 22 14	6.16% 5.69% 5.21% 3.31%	No	Concurrent use of metoclopramide and CNS depressants (e.g. sedatives, hypnotics, opiates, and anxiolytics) may result in increased risk of CNS depression and should be avoided. If coadministration is necessary, the possibility of adverse effects should be monitored [47]	Additive pharmacologic effects
Dexamethasone—tramadol	19	4.50%	No	Concurrent use of tramadol with a CYP3A4 inducers (such as dexamethasone) may result in reduced tramadol exposure. If concomitant use is needed, patients should be closely monitored for decreased efficacy or signs of opioid withdrawal syndrome, and an increase in the tramadol dose should be considered necessary. If a CYP3A4 inducer is discontinued, tramadol dose reduction should be considered, and patients should be closely monitored for signs of serotonin syndrome, respiratory depression, or sedation [48]	Induction of CYP3A4-mediated tramadol metabolism
Cisplatin—furosemide	16	3.79%	Yes	Concurrent use of cisplatin and furosemide may result in increased risk of ototoxicity and/or nephrotoxicity of cisplatin. Therefore, furosemide should be administered at lower doses and with a positive fluid balance when given for forced diuresis during cisplatin therapy. If cisplatin and furosemide are co-administered, monitoring renal and auditory function may be warranted [49, 50]	Additive or synergistic toxicity
Dexamethasone—doxorubicin	15	3.55%	Yes	Concurrent use of doxorubicin, a CYP3A4 substrate, with selected CYP3A4 inducers (e.g. dexamethasone) should be avoided, as reduced doxorubicin plasma concentrations may result [51]	Induction of CYP3A4-mediated doxorubicin metabolism
Morphine—ondansetron	14	3.31%	No	Concurrent use of opioids, such as morphine, with serotonergic drugs (e.g. ondansetron) may result in increased risk of serotonin syndrome. If concomitant use is needed, the patient must be carefully observed, particularly during treatment initiation and dose adjustments. Morphine must be discontinued if serotonin syndrome is suspected [52]	Additive serotonergic effects
Filgrastim—vincristine	12	2.84%	Yes	Severe atypical peripheral neuropathy was reported to occur significantly more commonly in patients with lymphomas receiving a colony stimulating factor (sargramostim or filgrastim) with vincristine than vincristine alone. Peripheral neuropathy is characterised by a constant severe, sharp, or burning pain confined to the feet. In patients receiving vincristine and filgrastim the total dose of vincristine used in the first cycle should be restricted and patients should be monitored carefully for symptoms of peripheral neuropathy [53]	Unknown
Ondansetron—oxaliplatin	9	2.13%	Yes	Concurrent use of oxaliplatin and QT interval prolonging drugs may result in increased risk of QT-interval prolongation and ventricular arrhythmias. Concomitant use of oxaliplatin and other drugs with a known potential to prolong the QT interval (ondansetron is here included here) must be avoided [54]	Additive QT-interval prolongation

*Abbreviations: DDIs* Drug–drug interactions, *SDIs* Severe drug interactions, *ANA* Antineoplastic agent, *CNS* Central nervous system

### Factors associated with polypharmacy and DDIs

**Fig. 3:**
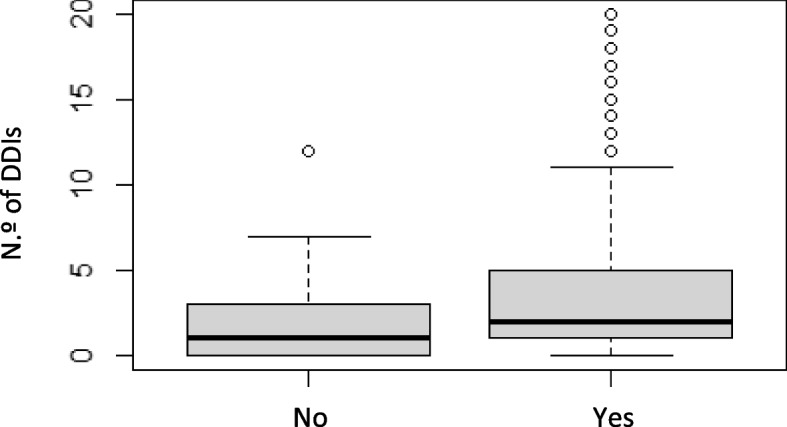
Boxplot of relationship between the number of DDIs and the existence chronic diseases

No significant association was detected between age and gender with polypharmacy, excessive polypharmacy, and potential DDIs (*p*-value > 0.05). It was possible to observe a statistically significant relationship between the existence of chronic diseases with excessive polypharmacy (*p*-value = 0.007) and with DDIs (medNo = 1.00 (0–12); medYes = 2.00 (0–20); *p*-value < 0.001) (*p*-value = 0.0006721) (Fig. 3).

**Fig. 4:**
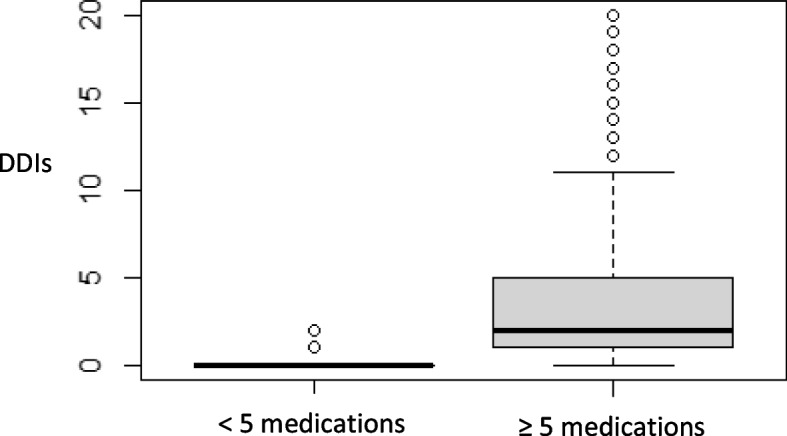
Boxplot of n.º of DDIs identified and polypharmacy

**Fig. 5:**
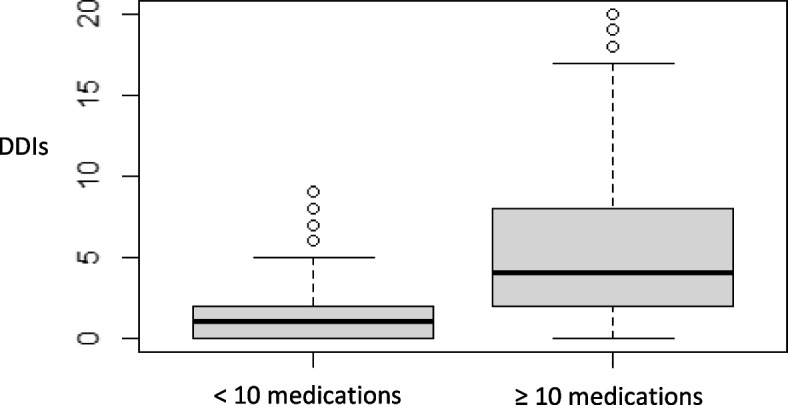
Boxplot of n.º of DDIs identified and excessive polypharmacy

Polypharmacy and DDIs use were significantly intercorrelated (med_<5_ = 0.00 (0–2); med_≥5_ = 2.00 (0–20); *p*-value < 0.001) (Fig. 4). The same happened with excessive polypharmacy (med_<10_ = 1.00 (0–9); med_≥10_ = 4.00 (0–20); *p*-value < 0.001) (Fig. 5) (Wilcoxon’s test; *p*-value < 2.2e-16).

In the group of patients with polypharmacy, 84.43% (*N* = 412) had at least one DDI, which was significantly higher when compared to the other patients. The same happened in the group with excessive polypharmacy (c^2^; *p*-value < 2.2e-16).

Regarding the patients’ profiles associated with the occurrence of polypharmacy, potential DDIs or SDIs, their relationship with the administration of high-risk medications, and/or the existence of comorbidities with cardiac risk were analysed (Table 5).

**Table 5:** Factors associated with polypharmacy, DDIs, and SDIs

Factor	Polypharmacy, n (%)			DDIs, n (%)			SDIs, n (%)		
	**0–4** **(*N* = 64)**	** ≥ 5** **(*N* = 488)**	***p* value**	**No** **(*N* = 130)**	**Yes** **(*N* = 422)**	***p* value**	**No** **(*N* = 242)**	**Yes** **(*N* = 310)**	***p* value**
**Age, years**									
Mean (SD)	71.6 (5.2)	71.9 (5.2)	0.671	71.8 (5.7)	71.9 (5.1)	0.877	72.0 (5.6)	71.8 (4.9)	0.592
**Sex**									
Male	35 (54.7)	267 (54.7)	1.000	72 (55.4)	230 (54.5)	0.939	139 (57.4)	163 (52.6)	0.293
Female	29 (45.3)	221 (45.3)		58 (44.6)	192 (45.5)		103 (42.6)	147 (47.4)	
**Comorbidities with cardiac risk**									
Hypertension	20 (31.2)	278 (57)	< 0.001	59 (45.4)	239 (56.6)	0.032	127 (52.5)	171 (55.2)	0.588
Diabetes mellitus	4 (6.3)	121 (24.8)	0.002	9 (7.0)	116 (27.5)	< 0.001	43 (17.9)	82 (26.5)	0.023
Dyslipidemia	18 (28.1)	197 (40.7)	0.072	47 (36.7)	168 (40.0)	0.574	85 (35.4)	130 (42.2)	0.127
**High-risk medication**	18 (28.1)	248 (50.8)	0.001	30 (23.1)	236 (55.9)	< 0.001	73 (30.2)	193 (62.3)	< 0.001

*Abbreviations*: *DDIs* Drug–drug interactions, *SDIs* Severe drug interactions

The administration of high-risk medications was found to be associated with a higher risk of polypharmacy (OR = 2.64 [1.51–4.79]; *p* < 0.05), DDIs (OR = 4.23 [2.72–6.73]; *p *< 0.001) and SDIs (OR = 3.82 [2.68–5.48]; *p* < 0.001), when compared to patients who did not take these drugs. Patients with hypertension and diabetes mellitus presented a higher possibility of polypharmacy (OR = 2.91 [1.69–5.19]; *p* < 0.001 and OR = 4.88 [1.96–16.32]; *p* = 0.003) and DDIs (OR = 1.57 [1.06–2.34]; *p* = 0.025 and OR = 5.01 [2.60–10.92]; *p* < 0.001) when compared with patients without these pathologies. Analyzing the SDIs, only individuals with diabetes mellitus presented a higher risk for the occurrence of this type of interaction, compared to patients without this pathology (OR = 1.65 [1.09–2.51]; *p* = 0.018).

## Discussion

Polypharmacy in older patients and the consequent increased potential for DDIs and SDIs can lead to several problems, such as toxicity, adverse drug events, and hospitalisation. Our study confirmed the high prevalence of polypharmacy, potential DDIs, and SDIs in older adults with a cancer diagnosis: 88.40% (*N* = 488), 76.45% (*N* = 422), and 56.16% (*N* = 310), respectively. Regarding ANAs, 40.76% (*N* = 225) of all enrolled patients had a risk of potential DDIs, and 21.20% (*N* = 117) were at risk of SDIs.

The high prevalence is similar to those obtained by Nightingale et al. [14] and Mohamed et al. (2018) for polypharmacy and by Beinse et al. [36], Popa et al. [35] and Leger et al. (2018) for DDIs. Different results were found in other studies, with lower prevalence rates, ranging from 30.8% to 77.6% for polypharmacy [13, 15, 16, 20, 26, 29, 37, 55] and from 30.6% to 57.9% for DDIs [37, 56]. Excessive polypharmacy was recorded in 44.57% of the patients (*N* = 246), similar to the results reported by Nightingale et al. [37]. Lower results have been described in other studies [13, 15, 16]. These differences might be related to diverse definitions of polypharmacy or to the DDI assessment tools used among the studies. In addition, the studies presented several differences considering the study design, methods, and a variety of clinical decision support instruments used.

Regarding polypharmacy, Popa et al. [35] reflected that has high values in the older population due to (i) the increase in chronic diseases, (ii) the absence of a primary care provider capable of coordinating the care of different specialties, (iii) the greater use of alternative forms of treatments, and (iv) the administration of unnecessary and/or duplicate medications when multiple physicians and multiple care sites are involved [35]. In Europe, which has high ageing rates, there is insufficient polypharmacy data in geriatric patients with cancer. Polypharmacy is an essential component of geriatric assessment and must be determined before starting chemotherapy [7, 42].

Beyond the patient’s usual medication, our study included data on chemotherapy treatment and supportive medications, which justifies the high prevalence of DDIs, and DDIs involving ANAs. Most studies assess the prevalence of DDIs using clinical decision support software, which should provide higher-quality information about DDIs. However, there is a lack of standardisation of the criteria for the classification of DDIs [57–59], which makes it difficult to compare different studies. Tools should be sensitive (identifying potential clinically important DDIs) and specific (avoiding the identification of DDIs of no clinical interest) [14]. In our study, we used Micromedex® because of its high sensitivity and specificity rated with oncology medications [44, 45]. The same tool has been used in other studies [14, 60].

Drug interaction software used in clinical decision support can be extremely helpful in the management of polypharmacy and its associated risks, especially when used in an integrated manner with medical records and electronic prescriptions. It is important to create objective and well-defined criteria that allow for the identification and classification of potential DDIs/SDIs in a coherent and consensual way. It is also relevant that the information available in the different tools is evidence-based and clinically relevant. The creation of universal databases is essential for a broader, more comprehensive, uniform, and rigorous knowledge of this problem.

Polypharmacy was significantly correlated with increased DDIs (Wilcoxon’s test; *p* < 0.001). This result might explain the increased risk of DDIs when taking more medication, especially in older adults receiving chemotherapy, which has a narrow therapeutic window. Moreover, older people have physiological and pharmacokinetic changes and are more vulnerable to concurrent medications [61, 62].

In addition to potential DDIs, SDIs were reported in 56.16% of patients (*N* = 310), similar to those identified by Lavan et al. [16] and Nightingale et al. [14], with 50.5% and 61.3%, respectively. Different values were reported by Popa et al. [35] (21.3%), Hong et al. [13] (30.6%), Alkan et al. [26] (35.1%), Mohamed et al. (2018) (70%) and Guven et al. [55] (85.7%). Such variation can be justified by differences in the definition of SDIs, the software used for detection, patients’ cancer types, and treatment protocols [13].

Overall, 40.76% (*N* = 225) of all enrolled patients had a risk of potential DDIs involving ANAs. The values are concordant with the results obtained by Popa, et al. [35], which registered 45.9%. Beinse, et al. [36] recorded lower values (26.4%) of DDIs involving ANAs. The proportion of DDIs involving ANAs (21.67%) was lower than reported by Popa et al. [35] (29.3%) and higher than what was reported by Beinse et al. [36] (13%), in the total of identified DDIs. In this study, SDIs involving ANAs were identified in 21.20% of patients (*N* = 117) and corresponded to 27.72% of patients with DDIs, values higher than those reported by Popa, et al. [35]. SDIs involving ANAs correspond to 10.40% of total registered DDIs, 18.51% of total SDIs, and 47.96% of total DDIs involving ANAs. The ANAs most frequently associated with SDIs were doxorubicin, cyclophosphamide, cisplatin, vincristine, and oxaliplatin. The most frequently identified SDIs involved ANAs or supportive medication in the oncological context. Greater awareness of occurrence of DDIs may lead to chemotherapy adjustments or the careful monitoring of side effects.

The administration of high-risk medications is important because they may be associated with an increase in adverse drug events and hospitalisation risks in the older population, therefore its use should be reviewed avoided, and potential drug-drug interactions should be discussed and avoided whenever possible [39, 40]. We observed that the administration of high-risk medications was associated with a higher risk of occurrence of the three conditions (polypharmacy, OR = 2.64; DDIs, OR = 4.23; and SDIs, OR = 3.82) compared to patients who did not take these drugs. However, Hong et al. [13] did not find any association between the use of those six high-risk medications and treatment toxicity, hospitalisation, or emergency room visits [13].

The occurrence of polypharmacy and DDIs was higher in patients who had hypertension (OR = 2.91; OR = 1.57) or diabetes mellitus (OR = 4.88; OR = 5.01) than in patients who did not present with these pathologies. The occurrence of SDIs was higher only in patients with diabetes mellitus, compared to patients without this pathology (OR = 1.65). Polypharmacy and DDIs were significantly associated with inappropriate drug prescriptions in older adult patients with cardiovascular diseases, highlighting the need for interventions to improve the practice of adequate prescription in these patients, carefully reviewing the medications and adjusting therapy to avoid adverse drug reactions and negative health outcomes [63, 64]. Polypharmacy and SDIs may be related to the risk of a reduced health-related quality of life in older adults with diabetes mellitus. Recommendations are important to simplify medication regimens by reducing the number of medications administered [65, 66].

Although patients with polypharmacy and DDIs may be at increased risk of hospitalisation or emergency room visits [13, 35, 36, 67], the effects of polypharmacy in patients undergoing chemotherapy are not consensual. Maggiore et al. [29] showed that polypharmacy had no impact on toxicity related to chemotherapy, emergency room visits, or hospitalisation in a geriatric oncology population. The causes of emergency room visits, or hospitalisation may be more complex and can be due to chemotherapy toxicity, symptoms from cancer itself and other cancer-related complications, presence, or worsening of comorbidities conditions, or all the above, which makes them more susceptible to complications and eventual hospitalisations [13, 15, 36]. Still, studies have highlighted the importance of considering DDIs in the management of older cancer patients to prevent adverse events and unplanned hospitalisations [35, 36].

This study contributes to raising health professionals´ awareness of the importance and risks associated with polypharmacy contexts, increasing concern for the safe administration of medication in these patients. Measures to reduce polypharmacy, and consequent DDIs/SDIs, involve promoting and triggering the implementation of adequate and consistent procedures, in an articulated and coordinated manner, at the time of prescription and/or intervention by the pharmacy professional and the physician (geriatric and oncology). Studies show that patient assessment and intervention involving pharmacy professionals is an effective strategy to reduce medication-related problems and optimise therapeutic treatments [37, 68–71].

Strategies to evaluate, review and simplify medication regimens are essential to decrease the risks of interactions and ensure patient safety. Medication reviews should be performed by professionals with experience in clinical pharmacology, the management of multimorbidity, and a clear knowledge of the oncological disease in this population. Therefore, an integrated, systematic, and standardised geriatric assessment is essential before starting a chemotherapy treatment. Deprescribing approaches and pharmacological monitoring should be discussed and performed to optimise therapeutic regimens and may be of value to avoid drug-related problems and allow for the continuity and success of the implemented treatment.

The present study has some limitations. As this is an observational study, in which data collection was also based on patients' reports, it is possible that not all administered drugs were identified (including over-the-counter drugs / non-prescription medications, alternative, and/or herbal products). Although it was conducted in three reference institutions, many patients of the identified were not included in the study for several reasons (e.g., confusing, incomplete, or incoherent information, and refusal of patients to participate), which may compromise the generalization of the results. Further, there was no standard of care. The patients were treated at the discretion of their physicians, which reinforces the importance of intervention involving a multidisciplinary geriatric oncology team. We analyse drugs only at the start of chemotherapy. There was no control during treatment related to drug compliance or of any adverse reactions that may have led to changes in the medication. The impact of the dosage and/or frequency of the medications was not investigated. Furthermore, our study was not designed as a prospective intervention study; therefore, it did not assess patient outcomes to identify the clinical consequences of polypharmacy or reported DDIs. Prospective studies allow assessment of the prevalence of clinically significant DDIs that require intervention. Further, studies dedicated to polypharmacy and drug-related problems are equally needed, as it is necessary to identify, characterize, and avoid the reasons for eventual hospitalisations or emergency room visits [60, 71, 72].

## Conclusion

Due to the ageing of the population and the increased prevalence of cancer with age, special attention should be given to geriatric cancer patients. More comorbidities and consequent polypharmacy and DDIs make caring for these patients particularly complex. This study confirms the high prevalence of polypharmacy, potential DDIs, and SDIs in older adults with cancer and the involvement of ANAs, resulting in critical concern in these patients. Polypharmacy was significantly correlated with increased DDIs. Overall, our findings indicate an additional burden on these older patients, especially those with cardiovascular risk factors (hypertension and diabetes mellitus) and those who administer high-risk medications.

In summary, the results obtained in this study indicate the need for further research and greater awareness of patients and professionals to identify, discuss and develop an intervention strategy based on a more careful prescription so that polypharmacy and DDIs/SDIs can be avoided whenever possible.

## Data Availability

The data underlying this article are available in the article. Any additional information is available from the corresponding author on reasonable request.
